# Therapeutic Plasma Exchange as a Treatment for Autoimmune Neurological Disease

**DOI:** 10.1155/2020/3484659

**Published:** 2020-07-31

**Authors:** Ivana Nieto-Aristizábal, Álvaro J. Vivas, Pablo Ruiz-Montaño, Cristian C. Aragón, Iván Posso-Osorio, Jairo Quiñones, Julián Alejandro Rivillas, Gabriel J. Tobón

**Affiliations:** ^1^GIRAT: Grupo de Investigación en Reumatología, Autoinmunidad y Medicina Traslacional, Universidad Icesi and Fundación Valle del Lili, Cra. 98 18-49, Cali, Colombia; ^2^Medical School, Universidad Icesi, Calle 18 No. 122-135, Cali, Colombia; ^3^Department of Neurology, Fundación Valle del Lili, Cra. 98 18-49, Cali, Colombia; ^4^Unit of Neuroimmunology, Fundación Valle del Lili, Cra. 98 18-49, Cali, Colombia

## Abstract

**Introduction:**

Therapeutic plasma exchange (TPE) is commonly used as treatment of certain autoimmune neurological diseases (ANDs), and its main objective is the removal of pathogenic autoantibodies. Our aim was to describe the clinical profile and the experience with the usage of TPE in patients with ANDs at our institution.

**Methods:**

This is an observational retrospective study, including medical records of patients with diagnosis of ANDs who received TPE, between 2011 and 2018. Characteristics of TPE, such as number of cycles, type of replacement solution, and adverse effects, were evaluated. The modified Rankin Scale (mRS) was applied to measure the clinical response after the therapy.

**Results:**

187 patients were included with the following diagnoses: myasthenia gravis (MG), *n* = 70 (37%); Guillain–Barré syndrome (GBS), *n* = 53 (28.3%), neuromyelitis optica spectrum disorders (NMOSD), *n* = 35 (18.7%); chronic inflammatory demyelinating polyneuropathy (CIDP), *n* = 23 (12.2%); and autoimmune encephalitis (AE), *n* = 6 (3.2%). The most used types of replacement solution were albumin (*n* = 131, 70%) and succinylated gelatin (*n* = 45, 24%). All patients received a median of five cycles (IQR 5-5). Hypotension and hydroelectrolytic disorders were the main complications. After TPE, 99 patients (52.9%) showed improvement in the mRS scores and a statistical significance (*p* < 0.05) was seen between the admission score and after TPE for every diagnosis except for CIDP.

**Conclusion:**

TPE has an adequate safety profile, and improvement in functionality in treated patients reflects its effectiveness.

## 1. Introduction

Therapeutic plasma exchange (TPE) is defined by the American Society for Apheresis (ASFA) 2019 guidelines as “A therapeutic procedure in which the blood of the patient is passed through a medical device which separates plasma from the other components of blood….”. Unlike plasmapheresis, TPE involves plasma removal and replacement with a solution such as a colloid solution (e.g., albumin and/or plasma) or a combination of a crystalloid/colloid solution [1].

Even though it was initially conceived as a treatment for hematological diseases with presumed or demonstrated immune pathophysiology [2], treatment using TPE has been extended to a variety of pathologies including kidney, autoimmune rheumatological, and neurological diseases, with the latter being the pathology most frequently treated by TPE [3]. The increased usage of TPE has likely followed an increased understanding of its mechanisms of action, which range from the removal of pathogenic autoantibodies and immune complexes to improvement in monocyte function [2]. Among the autoimmune neurological diseases (ANDs) treated using TPE, chronic inflammatory demyelinating polyneuropathy (CIDP), Guillain–Barré syndrome (GBS), myasthenia gravis (MG), neuromyelitis optica spectrum disorders (NMOSDs), and autoimmune encephalitis (AE) are well described. TPE is typically used alone or in conjunction with other treatment options, such as intravenous immunoglobulins (IVIG) and corticosteroids, as a first-line treatment for some of these disorders.

The great majority of descriptions of the clinical experience of TPE usage for this group of diseases are from North American or European cohorts [4–7]. In addition, environmental and genetic factors differ between populations, and these factors may influence the response the individuals have on different therapeutic interventions. Many of the mentioned ANDs have shown an exponential increase in occurrence in recent years [8–11]. Therefore, it is important to study in depth the safety and effectiveness of their available treatments, such as TPE. In this study, we aimed to describe the factors associated with indications, types of replacement solution, adverse effects, and treatment effectiveness of this intervention in patients with different ANDs.

## 2. Material and Methods

This was a retrospective and observational study conducted at Fundación Valle del Lili, a high-complexity center in Cali, Colombia. The medical records of patients with ANDs diagnosed by neurologists, who received at least one TPE in our institution between 2011 and 2018, were included in our analyses. In each case, the number of treatment cycles, type of replacement solution, clinical characteristics as duration of inpatient stay, adverse effects, and death of patients were evaluated. For identifying relapses, admissions due to ANDs up to one year after TPE were quantified.

TPE was prescribed by neurologists and was always administered in the intensive care unit (ICU) by trained nurses. The procedure comprised centrifugation, where prophylactic calcium was not routine. In each case, according to TPE characteristics (e.g., volume, replacement solution), anticoagulation with citrate and replacement solutions were administered in the proportions of 1 : 12 to 1 : 16.

Since most of the evaluated diagnoses (e.g., MG, GBS, and CIDP) have a disease-specific scale, we used the modified Rankin Scale (mRS) to measure and compare the clinical improvement between our patients. Although initially used for stroke treatment, it has also been applied in several different contexts including the evaluation of outcomes in NMOSD [12], AE [13, 14], MG [15], CIDP [16], GBS [17], and other neurological conditions [18].

Thus, the mRS was applied to every patient at three different times: at admission, using the admission note of the department of neurology; after TPE, using their progress note registered after completing all the cycles; and 90-days after receiving TPE, using medical records of follow-up appointments with neurologists. mRS measurement was performed by the research group with a previous training by a neurology resident.

The mRS was first introduced in 1957 for measuring outcomes in acute stroke and was modified to its seven-grade version as follows: 0, no symptoms; 1, no significant disability; 2, slight disability; 3, moderate disability; 4, moderately severe disability; 5, severe disability; and 6, dead [19, 20]. The proposed interpretation of the mRS is based on two ranges of scores, 0–2 and 3–6, which are defined as favorable and nonfavorable, respectively; however, when looking at scores as individual points, changes in a single one have been stated to be clinically relevant [21].

Statistical analyses were performed using STATA v.14® (StataCorp LP, College Station, TX). Quantitative variables are shown as means and medians with standard deviations (SD) and interquartile ranges (IQRs) according to their distribution on the basis of the Shapiro–Wilk test. Quantitative variables are also shown with frequencies and proportions. mRS scores are presented as medians with IQRs; scores at admission and after TPE were compared with the Wilcoxon matched-pair signed-rank test.

## 3. Results

A total of 187 patients were included in this study. Most of them were women (*n* = 104, 55.6%), and the median age at admission was 50 (IQR 32–64) years. The most frequent AND was MG, with 70 cases (37.4%), followed by GBS, NMOSD, CIDP, and AE (Table 1).

TPE was performed using the centrifugation method, and a catheter was placed at the jugular (*n* = 166, 88.7%), femoral (*n* = 18, 9.6%), or subclavian (*n* = 3, 1.6%) positions.

The replacement solutions used in TPE were albumin, fresh frozen plasma, and succinylated gelatin. Albumin alone was used in most patients (*n* = 131, 70.5%), whereas albumin in combination with fresh frozen plasma was used in one patient only. In the general sample, the median number of TPEs per patient was five (IQR 5-5) (Table 1). Separated by diagnosis, it was the same in MG, AE, and CIDP; in NMOSD and GBS, the median remained five with a range slightly wider (IQR 5-6). All of the TPEs were indicated daily. The mean exchanged volume was 2278 mL (SD 525.7), and the mean TPE time was 67 minutes (SD 17.5).

Patients had a median of inpatient stay of 12 (IQR 8–19) days. Those with AE had the longest of all, with a median of 24.5 (IQR 17–49) days; this group also required more days in the ICU than other groups. The complications observed after TPE was provided were as follows: 106 (56.6%) patients presented with hypotension, 102 (54.4%) presented with electrolytic disorders, and 85 (45.4%) presented with some complications associated with the Mahurkar catheter (other complications were found at a lesser extent). The presence of infection was evaluated during the 14 days following TPE: a total of 21 (11.2%) cases were observed, of which the majority were patients with GBS (*n* = 10, 47.6%). Infections were subdivided on the basis of the causative agent, with bacteria as the principal factor (*n* = 17/21, 80.9%); viral infections occurred in two (9.5%) patients, fungal infection in two (9.5%), mycobacterial infection in one (4.8%), and parasitic infection in one (4.8%). The most common pathogen was *E*. *coli*, with 4 cases (19%), followed by *P*. *mirabilis* and *S*. *aureus*, both with 3 cases (14.3%). Two individuals had two agents of infection at the same time (Table 2).

Three (1.6%) patients died during hospitalization due to causes associated with their neurological disease and septic shock. Clinical relapse in the year following TPE was measured, and the largest proportion of relapses occurred in patients with MG (Table 2).

On the basis of the mRS score for each patient at admission and after TPE, 99 (52.9%) patients showed decreased scores after TPE, 67 (35.8%) showed no difference, and 21 (11.2%) showed increased scores after therapy. Although three patients died, two were receiving TPE at the time of death and the other one had finished TPE and remained alive for three additional months, which explains the two scores of “6” in the final measurement (Table 3). In addition, a statistically significant difference was noted between the median mRS scores before and after TPE in general (*p*=0) and for each disease: MG, *p*=0; GBS, *p*=0.0019; NMOSD, *p*=0.004; AE, *p*=0.0516. CIDP was the only one in which the difference was not significant (*p*=0.2019) (Table 4).

We calculated the mean/median time patients took to present relapses depending on their mRS after TPE and 90 days after TPE. It is important to notice that only 104 patients have a calculated mRS 90 days after TPE due to absence of medical records in that specific time (Table 5).

On the other hand, we calculated the difference in points between the mRS before and after TPE (the “Delta TPE”) and looked for any interrelation with relapse time, but we did not find any.

## 4. Discussion

This is a real-life study that shows the experience with the usage of TPE in multiple ANDs in a big cohort of 187 patients who were treated at a high-complexity center in Colombia. In 2019, the ASFA guidelines [1] defined specific recommendations for the use of TPE, and GBS, CIDP, MG, and NMOSD were included among the diseases of interest. Of these diseases, the strongest grades of recommendation were assigned to MG and CIDP, whereas the weakest grades were assigned to NMOSD and GBS [1]. Furthermore, neurological conditions in general are known to account for 44% of the cases in which TPE is indicated [22].

As previously discussed, TPE is as effective as IVIG in the treatment of GBS, CIDP, and MG; therefore, the decision to choose one or another treatment depends, in most cases, on the clinician's experience, treatment availability, and expenses. What can vary is the scheme in which the treatment is provided upon diagnosis [23]. In the present study, TPE was indicated in every patient due to the severity of their disease or therapeutic failure of other therapies, with MG being the most frequently observed disease (similar to previous reports) along with GBS [22]. In our center, TPE, rather than IVIG, is the preferred management option for ANDs, and it achieves favorable results.

Generally, the number of treatment cycles was approximately five, consistent with descriptions that report a total fluid exchange divided five times in MG [24], GBS [22], and CIDP [3, 25]. Jiao et al. reported a slightly different range, 2–7 treatment cycles, in 29 patients with NMOSD [26], and Moser et al. reported a wider range of cycles, 3–13, in 12 patients with AE [27].

In the analyses of the complications, hypotension occurred in approximately half of our patients even though a replacement solution was provided with TPE to prevent this outcome; this differs to apheresis where there is no fluid replacement [2]. Nonetheless, hypotension is common when using TPE for different indications [27–31]. Hydroelectrolytic disorders were the second-most frequent adverse reaction, not only in our study but also in the study by Clarck et al., who found that 59 of their patients required the replacement of at least one electrolyte after TPE (primarily for potassium and magnesium abnormalities) [30]. In our patients, 11.2% presented with infections, of which 80.9% were caused by bacteria. This finding is similar to a group studied by Lemaire et al., who reported infections caused primarily by bacteria, followed by viruses and fungus [29]. Severe adverse reactions, such as hypersensibility reactions and arrhythmias, were seen at a lower frequency in our group.

Lemaire et al. reported 50 patients who were admitted to the ICU, with 40% admitted due to neurological causes. In their cohort, the median inpatient stay was 20 (IQR 12–35) days, which was similar to our findings (20 days, IQR 8–19), whereas they reported a mortality proportion of 8% compared with 1.6% in our group [29]. As has been previously stated, two of the deaths in our study occurred before TPE was completed; thus, in some cases, these treatment options are given to critical patients in whom a clinical response may be difficult to achieve [30].

Clinical responses after TPE have been measured in different ways among several studies. For instance, Morgan et al. clinically evaluated the improvement in patients with NMO, concluding that there was a moderate improvement in functional scales [32]. For MG, CIDP, and GBS, Momtaz et al. focused on muscle strength, finding that of 31 patients with MG, 83.8% achieved a complete response; of 8 patients with CIDP, 87.5% achieved such a response; and of 39 patients with GBS, 51.3% responded in this way [25]. Titulaer et al. described a cohort of 577 patients diagnosed with AE, of whom 461 received first-line therapies, including TPE: 97% maintained mRS scores of 0–2 during a year of follow-up [33].

In this way, what we identify as the strength of our study is that we could compare multiple ANDs and bring objective data regarding clinical improvement after TPE with the application of the mRS, based on the experience of different authors who evaluated these diseases separately. It is to remark that although mRS is not a disease-specific scale for the diagnoses of interest, it is one that allows an approach in a more unified way among them. Then, similarly to the studies previously mentioned, 52.9% of our patients showed an improvement in their mRS scores whereas 35.8% did not show any response to treatment. Although no significant difference was noted between mRS scores before and after TPE in patients with CIDP, a decrease in median scores for each disease was observed in all diagnoses.

We did not find a pattern regarding time until relapses in relation to the mRS scores, but in the patients with a score of “0” at 90-days after TPE, it took the longest period of time to present a relapse, as would be expected. Then, it is reasonable to think that other factors may be associated with a better outcome following TPE, and it may be useful to consider these when making treatment decisions or considering expectations. In NMOSD, these factors include anti-AQP4 seronegativity (patients who are seronegative for anti-AQP4 but seropositive for anti-myelin oligodendrocyte glycoprotein generally present better outcomes) [12] and combination treatments with immunosuppressants [34]. In GBS, additional factors include the number of exchanges on the basis of the severity of disease [35] and the patient's age [17]. An efficient diagnosis approach [14] and early therapy initiation are common characteristics in various studies [12, 14, 33, 35, 36].

## 5. Conclusion

We showed TPE to have a high tolerance and strong safety profile in various ANDs. Furthermore, the improvement in mRS scores reflects the effectiveness of TPE in the treatment of MG, GBS, NMOSD, and AE.

## 6. Study Limitations

This study was retrospective in nature, which is why the measurements of clinical response had to be evaluated using a nonspecific scale. Furthermore, the scores used depend on information that was registered on medical records, which may lower the accuracy of the scores.

Additionally, the number of patients in each diagnosis limits the comparison between ANDs. The small number of patients with AE must be considered when interpreting the results. Finally, not every patient went for outpatient control visit 90-days after TPE, diminishing the study sample at that time, thus limiting the evaluation and/or comparison of variables such as mRS 90 days after TPE.

## Figures and Tables

**Table 1:** Demographic and TPE characteristics.

Characteristics	*N* (%)
Age^*∗∗*^	50 (32–64)

*Sex*	
Female	104 (55.6)
Male	83 (44.4)

*Autoimmune neurological disease*	187 (100)
MG	70 (37.4)
GBS	53 (28.3)
NMOSD	35 (18.7)
CIDP	23 (12.3)
AE	6 (3.2)

*Types of replacement solutions*	
Albumin	131
Succinylated gelatin	45
Albumin + succinylated gelatin	10
Albumin + fresh frozen plasma	1
*Number of TPEs by patient * ^*∗∗*^	5 (5–5)

^*∗∗*^Median (IQR). MG, myasthenia gravis; GBS, Guillain–Barré syndrome; NMOSD, neuromyelitis optica spectrum disorders; CIDP, chronic inflammatory demyelinating polyneuropathy; AE, autoimmune encephalitis. TPE, therapeutic plasmatic exchange.

**Table 2:** Outcomes related to TPE.

	General,*n* = 187	MG,*n* = 70	GBS, *n* = 53	NMOSD,*n* = 35	CIDP,*n* = 23	AE, *n* = 6
*Days of inpatient stay*, *median* (*IQR*)						
Inhospital stay	12 (8–19)	11 (7–15)	13 (9–33)	12 (8–18)	10 (7–16)	24.5 (17–49)
ICU stay	7 (6–12)	7 (6–10)	9 (6–24)	6 (6–9)	6 (6–8)	10 (6–15)

*Complications*, *n* (*%*)						
Hypotension	106 (56.6)	35 (50)	35 (66)	17 (48.5)	15 (65.2)	4 (66.6)
Hydroelectrolytic disorders	102 (54.5)	35 (50)	30 (56.6)	23 (65.7)	11 (47.8)	3 (50)
Related to Mahurkar catheter	85 (45.4)	29 (41.2)	26 (49)	15 (42.8)	12 (52.1)	3 (50)
Bleeding	56 (29.9)	22 (31.4)	19 (35.8)	9 (25.7)	4 (17.3)	2 (33.3)
Infections	21 (11.2)	5 (7.1)	10 (18.8)	4 (11.4)	2 (8.6)	0
Thrombosis	4 (2.1)	1 (1.4)	3 (5.6)	0	0	0
Hypersensibility	2 (1)	1 (1.4)	0	0	0	1 (16.6)
Arrhythmias	2 (1)	1 (1.4)	0	1 (2.8)	0	0
Hemolysis	0	0	0	0	0	0

*Death during hospitalization*, *n* (*%*)						
	3 (1.6)	0	1 (1.8)	0	2 (8.6)	0

*Relapse during the year after*, *n* (*%*)						
Number of patients	59 (31.5)	33 (47.1)	3 (5.6)	14 (40)	7 (30.4)	2 (33.3)

MG, myasthenia gravis; GBS, Guillain–Barré syndrome; NMOSD, neuromyelitis optica spectrum disorders; CIDP, chronic inflammatory demyelinating polyneuropathy; AE, autoimmune encephalitis IQR, interquartile range; ICU, intensive care unit.

**Table 3:** Number of patients in each category of the modified Rankin Scale before and after TPE.

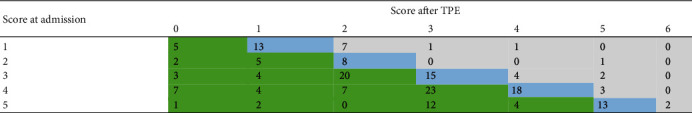

Each cell shows the number of patients who had a specific score at admission and after TPE. In green, patients who had a reduction in the scores after TPE; in blue, patients in whom the scores remained equal; in gray, patients who had an increase in the scores.

**Table 4:** Modified Rankin Scale scores before TPE compared with scores after TPE.

Diagnosis^*∗∗*^	Before TPE	After TPE	*p* value
General	4 (3–4)	3 (2–4)	**0**
MG	3 (2–4)	1 (1–2)	**0**
GBS	4 (3–5)	3 (3–4)	**0.0019**
NMOSD	4 (3–4)	3 (2–4)	**0.004**
CIDP	4 (3–4)	3 (2–4)	0.2019
AE	4 (3–5)	3 (2–3)	**0.0516**

^*∗∗*^Median (IQR), Wilcoxon matched-pairs signed-ranks test.

**Table 5:** Time until relapses according to mRS scores after TPE and 90 days after TPE.

Relapses <90 days after TPE				Relapses >90 days after TPE			
mRS score after TPE	Patients with the score (*n* = 185)	Patients who relapsed, *n* (%)	Time until relapse (days)	mRS score 90 days after TPE	Patients with the score (*n* = 104)	Patients who relapsed, *n* (%)	Time until relapse (days)
5	19	1 (5,55)	71	5	1	1 (100)	195
4	27	2 (7,4)	34,5 (26-43)^*∗∗*^	4	15	4 (26.7%)	139,5 (115,25-166)^*∗∗*^
3	51	8 (15,7)	12,5 (4,25–47,75)^*∗∗*^	3	30	7 (23%)	151 (29,35)^*∗*^
2	42	7 (16,7)	50 (30,04)^*∗*^	2	28	5 (17,86%)	160,2 (69,79)^*∗*^
1	28	6 (22,2)	24,6 (15,9)^*∗*^	1	22	5 (22,7%)	201,2 (94,73)^*∗*^
0	18	4 (22,2)	16,5 (4,75–56,75)^*∗∗*^	0	8	1 (12,5%)	272

^*∗*^Mean (SD); ^*∗∗*^Median (IQR). mRS, modified Rankin Scale.

## Data Availability

The data used to support the findings of this study are available from the corresponding author upon request.
